# Cortical thickness as predictor of response to exercise in people with Parkinson's disease

**DOI:** 10.1002/hbm.25211

**Published:** 2020-10-09

**Authors:** Carla Silva‐Batista, Anjanibhargavi Ragothaman, Martina Mancini, Patricia Carlson‐Kuhta, Graham Harker, Se Hee Jung, John G. Nutt, Damien A Fair, Fay B. Horak, Oscar Miranda‐Domínguez

**Affiliations:** ^1^ Exercise Neuroscience Research Group University of São Paulo SP Brazil; ^2^ Department of Neurology Oregon Health & Science University Portland Oregon USA; ^3^ Department of Rehabilitation Medicine Seoul National University Boramae Medical Center Seoul Republic of Korea; ^4^ Department of Behavioral Neuroscience Oregon Health & Science University Portland Oregon USA; ^5^ Veterans Affairs Portland Health Care System (VAPORHCS) Portland Oregon USA

**Keywords:** challenging exercise, dual‐task cost, freezing of gait, fronto‐parietal cortical, gray matter atrophy, visual cortical

## Abstract

We previously showed that dual‐task cost (DTC) on gait speed in people with Parkinson's disease (PD) improved after 6 weeks of the Agility Boot Camp with Cognitive Challenge (ABC‐C) exercise program. Since deficits in dual‐task gait speed are associated with freezing of gait and gray matter atrophy, here we performed preplanned secondary analyses to answer two questions: (a) Do people with PD who are freezers present similar improvements compared to nonfreezers in DTC on gait speed with ABC‐C? (b) Can cortical thickness at baseline predict responsiveness to the ABC‐C? The DTC from 39 freezers and 43 nonfreezers who completed 6 weeks of ABC‐C were analyzed. A subset of 51 participants (21 freezers and 30 nonfreezers) with high quality imaging data were used to characterize relationships between baseline cortical thickness and delta (Δ) DTC on gait speed following ABC‐C. Freezers showed larger ΔDTC on gait speed than nonfreezers with ABC‐C program (*p* < .05). Cortical thickness in visual and fronto‐parietal areas predicted ΔDTC on gait speed in freezers, whereas sensorimotor‐lateral thickness predicted ΔDTC on gait speed in nonfreezers (*p* < .05). When matched for motor severity, visual cortical thickness was a common predictor of response to exercise in all individuals, presenting the largest effect size. In conclusion, freezers improved gait automaticity even more than nonfreezers from cognitively challenging exercise. DTC on gait speed improvement was associated with larger baseline cortical thickness from different brain areas, depending on freezing status, but visual cortex thickness showed the most robust relationship with exercise‐induced improvements in DTC.

## 1 INTRODUCTION

Gait disturbances are common in Parkinson's disease (PD) and may be either continuous, such slow gait speed and reduced stride length, or intermittent, such as freezing of gait (FoG), in nature. FoG is defined as a brief, episodic absence, or marked reduction of forward progression of the feet despite the intention to walk (Nutt et al., 2011). Gait disturbances are typically aggravated during dual‐task walking (Woollacott & Shumway‐Cook, 2002). A dual‐task involves the simultaneous execution of two tasks, that have distinct goals such as walking and a cognitive task (Woollacott & Shumway‐Cook, 2002). Loss of automatic motor control (Wu & Hallett, 2005, 2008) and alterations in attentional control in PD (one's ability to divide and switch attention between tasks) (Wu & Hallett, 2005, 2008) might explain deficits in dual‐task gait. Thus, interventions aimed to restore gait automaticity might lead to significant improvements in dual‐task walking in people with PD.

A growing number of randomized, controlled trials (Conradsson et al., 2015; Lofgren, Conradsson, Rennie, Moe‐Nilssen, & Franzen, 2019; Strouwen et al., 2017) have demonstrated benefits of dual‐task training in PD. Our group also showed that the Agility Boot Camp with Cognitive Challenge (ABC‐C) exercise program, which includes dual‐task training (gait/balance and cognitive challenges simultaneously) improved dual‐task cost on gait speed (measure of relative change in gait speed between dual‐task trial relative to a single‐task trial) in people with PD with and without FoG (freezers and nonfreezers, respectively) (Jung et al., in press). We also demonstrated improvement in the Attention Network functional connectivity in a small subset of people with PD who are freezers comparing neuroimaging before and after the 6‐week ABC‐C program (King et al., 2020). However, we did not systematically investigate differences in responsiveness of dual‐task cost on gait speed in freezers and nonfreezers following the ABC‐C exercise program.

Our first hypothesis was that freezers would show a smaller improvement in dual‐task cost on gait speed than nonfreezers following the ABC‐C program. Freezers may not improve their dual‐task cost on gait speed following exercise as much as nonfreezers for three reasons. First, freezers have less effective motor‐learning capacities (Paul, Dibble, & Peterson, 2018). Second, freezers show an exaggerated loss of gait automaticity indicated by a higher dual‐task cost on gait speed compared to nonfreezers (de Souza Fortaleza et al., 2017). Third, deficits in dual‐task walking are correlated with more severe FoG (Spildooren et al., 2010), which may limit a large improvement in dual‐task cost on gait speed after exercise training for people with more severe FoG.

Our second hypothesis was that cortical thickness in visual and fronto‐parietal cortical areas at baseline would predict improvement in dual‐task cost on gait speed following the ABC‐C program in freezers (Piramide et al., 2020; Shine et al., 2013). The depth of the cortical gray matter layer that covers the surface of the brain, referred to as cortical thickness, has been extensively assessed in PD (Hanganu et al., 2013; Jubault et al., 2011; Mak et al., 2015; Pereira et al., 2012). Widespread cortical thinning has been reported in frontal, parietal, temporal, and occipital cortices in people with PD compared to healthy control people (Mak et al., 2015; Pereira et al., 2012). Abnormalities in visual and fronto‐parietal regions are more affected in freezers than nonfreezers, and reduced functional connectivity of the fronto‐parietal and visual regions are related to FoG severity (Canu et al., 2015; Tessitore et al., 2012). In addition, abnormal fronto‐parietal cortical activity (Herman, Rosenberg‐Katz, Jacob, Giladi, & Hausdorff, 2014; Shine, Naismith, & Lewis, 2011) and gray matter atrophy in fronto‐parietal areas (Kostic et al., 2012) are associated with FoG severity. Gray matter atrophy in the inferior parietal lobe is significantly reduced in freezers compared to nonfreezers and the atrophy is associated with slower gait speed under dual‐task conditions in PD (Herman et al., 2014). In dual‐task conditions, motor and cognitive tasks compete for similar cortical resources (Wu & Hallett, 2005, 2008). Thus, cortical thickness may have a key role in predicting improvement in dual‐task cost on gait speed following an exercise intervention.

Here, we aimed to answer two questions: (a) Do people with PD who are freezers show similar improvement in dual‐task cost on gait speed with ABC‐C as nonfreezers? (b) Can cortical thickness at baseline predict responsiveness to the ABC‐C program in dual‐task cost on gait speed improvement in people with PD?

## 2 METHODS

### 2.1 Participants

All people with PD were diagnosed by movement disorders specialists as having idiopathic PD based on the United Kingdom Brain Bank criteria (Hughes, Daniel, Kilford, & Lees, 1992). All people with PD were eligible if they were: (a) 50–90 years of age; (b) mild to moderate PD (Hoehn and Yahr Levels II–III); (c) on stable antiparkinsonian medication; (d) without major musculoskeletal, peripheral or central nervous system disorders (other than PD) that could significantly affect their balance and gait; (e) without excessive use of alcohol or recreational drugs; (f) without history of structural brain disease, active epilepsy, stroke or dementia that would interfere with consent or ability to follow testing procedures; (g) able to stand or walk for 2 min without an assistive device; (h) without a medical condition that precludes exercise; and (i) without claustrophobia, severe tremor, or any health history that would interfere or put the subject at risk near the powerful magnetic field of the MRI scanner (i.e., implanted devices and deep brain stimulation). Subjects were excluded if they could not stand or walk independently for two reasons: (a) to make sure that they could perform the walking trials during single and dual tasks to obtain valid outcome measures and (b) to be sure they would be safe in our challenging exercise program in a group setting. FoG was assessed by J.G.N. (a trained neurologist) who defined freezers as >0 on the New Freezing of Gait Questionnaire (NFoGQ) (Nieuwboer et al., 2009). Motor severity was assessed using Movement Disorders Society Unified Parkinson's Disease Rating Scale motor subscale scores (MDS‐UPDRS‐III) (Goetz et al., 2008). Postural instability and gait difficulty (PIGD) subscore was calculated to assess gait and balance (Dewey et al., 2014). The Montreal Cognitive Assessment (MoCA) was used to measure global cognition (Nasreddine et al., 2005). People with PD were assessed in the practical OFF levodopa state (12 hr withdrawal of antiparkinson medication). In addition, medication was kept stable during the study.

### 2.2 Study design and randomization

This was a secondary analysis of a randomized, single‐blinded, cross‐over exercise trial at Oregon Health & Science University (OHSU) and the Veterans Affairs Portland Health Care System (VAPORHCS). In this secondary analysis, the dual‐task cost on gait speed improvement and cortical thickness are secondary dependent and independent variables, respectively, as they represent a subset of the variables of a larger clinical trial (King et al., 2015). In the clinical trial (King et al., 2015), participants were randomized into one of two intervention groups, exercise first (ABC‐C) or education first, by a computerized block randomization (Research Electronic Data Capture, REDCap). Randomization was implemented by an independent statistician using a block size of 12 people (six in each program). After randomization, the exercise trainer (unblinded) notified the subjects by phone. Participants randomized to exercise first participated in a 6‐week ABC‐C program and crossed over to receive the 6‐week education program (education second), and individuals in education first participated in an education class and crossed over to receive ABC‐C program (exercise second).

Overall, 86 people with PD completed the study (Jung et al., in press). As here we are interested in the improvement in dual‐task cost on gait speed with exercise program, we used only dual‐task cost on gait speed of the 82 participants who completed the ABC‐C program (39 freezers [*n* = 23 for exercise‐first and *n* = 16 for exercise‐second] and 43 nonfreezers [*n* = 21 for exercise‐first and *n* = 22 for exercise‐second]). Four participants were excluded because they had two out of three assessments. In addition, no order (exercise‐first vs. education‐first) and period (sequence of assessments) effects were previously found in this cohort (Jung et al., in press).

To identify associations between cortical thickness and response to exercise, we used a subset (21 freezers and 30 nonfreezers) of these 82 participants who had high quality magnetic resonance imaging (MRI) at baseline to predict exercise response using dual‐task cost on gait speed improvement. Analyses were repeated after matching freezer (*n* = 21) and nonfreezer (*n* = 21) participants on motor severity using MDS‐UPDRS‐III in order to avoid our analysis to be biased by disease severity.

The OHSU and VAPORHCS ethics committees approved all aspects of the study (OHSU/VAPORHCS IRB protocols 4131 and 8979, respectively). All research was in compliance with the Helsinki Declarations. This trial was registered at clinical trials.gov (NCT02231073) and the trial protocol has been published (King et al., 2015).

### 2.3 ABC‐C program

The ABC‐C program was an adapted version of our ABC program (King et al., 2013). People with PD participated in an 80‐min, group (3–6 per group) exercise session for 3 days per week for 6 weeks. The exercises are designed as a circuit to challenge cognitive‐mobility skills known to be impaired in PD. Stations included: (a) Gait training, (b) PWR!Moves®, (c) Agility obstacle course, (d) Lunges, (e) Boxing, and (f) Adaptive Tai Chi. Each activity was chosen for its inherent focus on multidirectional movements, dynamic postural transitions, axial mobility, big movements and whole‐body motor sequencing. Each station was engaged for 10–20 min with rest periods in between stations, as needed. Each activity was systematically progressed from beginning to intermediate to advanced levels with cognitive challenges: (a) divided attention with secondary cognitive tasks, (b) response inhibition such as go‐no go commands and switching commands quickly, (c) set‐switching, (d) limiting visual cues, (e) increasing speed and resistance. The exercise class structure and progressions were led by a trained and experienced exercise trainer with oversight from a licensed physical therapist. Exercises are divided into three levels to provide an incremental progression of various activity tasks, with increasing levels of motor or cognitive challenge. Each activity was systematically progressed from beginning to intermediate to advanced levels by challenging with: (a) dual‐tasking by divided attention with secondary cognitive tasks, (b) response inhibition such as go‐no go commands and switching commands quickly, (c) limiting visual cues with focus on self‐initiated movements, (d) increasing the length, complexity, and novelty of whole body movement sequences, and (e) increasing repetitions, speed, amplitude, resistance, or balance requirements. Both the balance and cognitive challenge levels progressed in each station as tolerated by each individual and was recorded by the trainer. An individual was progressed when the trainer determined they were safely and accurately performing the exercise. Trained research assistants stood nearby and assisted participants who self‐reported frequent falls or had observed instability during the first exercise session.

### 2.4 Procedures

#### 2.4.1 Dependent variable

The participants were blinded to our hypothesis and expected outcomes. The researchers who performed baseline, midpoint, and final assessments of dual‐task cost on gait speed remained blinded to group assignment throughout the duration of the study and they did not help with the exercise sessions. The improvement in dual‐task cost was calculated as the percent difference of the dual‐task cost on gait speed values using midpoint—baseline for the exercise‐first, and final—midpoint for exercise‐second. Dual‐task cost on gait speed (dual‐task cost = [dual‐task – single task]/single‐task × 100) was measured on two self‐paced, walking trials: (a) 2‐min trial with no added cognitive task (single‐task), and (b) 1‐min trial with a simultaneous cognitive task (dual‐task) to not cause mental fatigue. In both conditions, people with PD were instructed to walk at a comfortable pace back and forth continuously between two lines 7.6 m apart. In the dual‐task condition, people with PD were instructed to walk while reciting every other letter of the alphabet. The order of the conditions was not randomized; the single‐task condition was always completed before the dual‐task condition and the single‐task condition was always completed after performing the cognitive test for 1 min while seated. Eight inertial measurement units (Opals, APDM, Inc., Portland) were placed on the feet, shins, lumbar (L5), sternum, and wrists by Velcro bands to measure gait. Gait speed (m/s) was quantified as average stride velocity from the Opal sensors for each trial. The Opal sensor includes triaxial accelerometers, gyroscopes and magnetometers and records signal data at 128 Hz. Inertial sensor data were wirelessly transferred to a laptop for automatic generation of gait measures by APDM proprietary software Mobility Lab 2 (Morris et al., 2019). Accuracy of the cognitive task while walking, compared to sitting, has been previously published (Jung et al., in press).

#### 2.4.2 Independent variable

For each individual with high quality MRI (e.g., without motion artifacts), we used as the independent variable, the brain cortical thickness measured at baseline to predict dual‐task cost on gait speed improvement.

##### MRI data acquisition

MRI assessments were obtained within the week before beginning the exercise program. Imaging data were acquired using two different scanners, a Siemens Trio 3T and, later, a Siemens Prisma 3T scanner with a 12‐channel head coil at the OHSU Advanced Imaging Research Center (AIRC). Data were collected by a trained and certified scanning technician and images were reviewed by a neurologist in the team if suspected anatomical abnormalities were found. Subjects were instructed to lie still and keep their eyes open. Head padding was provided to help subjects keep their heads still, earplugs were used to protect against scanner noise, and a leg bolster was provided to assist with back comfort. Depending on the participants' tolerance, weighted bags were also placed on the participants arms and legs if they thought this would be helpful in minimizing motion. High‐resolution, structural T1‐ and T2‐weighted images were obtained using the following parameters: Trio: T1‐weighted images were acquired using a sagittal magnetization prepared rapid gradient echo sequence: TR = 2,300 ms, TE = 3.58 ms, voxel size = 1 mm × 1 mm × 1.1 mm, slices = 160; T2‐weighted images were acquired using the following parameters: TR = 3,200 ms, TE = 497 ms, resolution *=* 1 mm isotropic, slices = 160; respectively. Prisma: T1‐weighted images were acquired using a sagittal magnetization prepared rapid gradient echo sequence: TR = 2,500 ms, TE = 2.88 ms, TI = 1,060 ms, resolution = 1 mm isotropic; T2‐weighted images were acquired using the following parameters: TR = 3,200 ms, TE = 565 ms, resolution *=* 1 mm isotropic, slices = 176; respectively. Diffusion field maps were also acquired to correct for geometric distortions caused by susceptibility artifact.

##### Preprocessing of baseline imaging data

Data were processed using a modified version of the workflow pipelines from the Human Connectome Project (DCAN‐Labs., 2019; Gilat et al., 2018; Glasser et al., 2013; Miranda‐Dominguez et al., 2018) (available in github at https://github.com/DCAN-Labs/abcd-hcp-pipeline), which include the use of FMRIB Software Library (FSL) (Jenkinson, Beckmann, Behrens, Woolrich, & Smith, 2012; Smith et al., 2004; Woolrich et al., 2009) and FreeSurfer tools (Dale, Fischl, & Sereno, 1999; Desikan et al., 2006; Fischl & Dale, 2000). Briefly, T1‐weighted and T2‐weighted volumes were first aligned to the MNI's AC‐PC axis and then nonlinearly normalized to the MNI atlas. This original alignment was refined using boundary based registration (Greve & Fischl, 2009). Then, optimally‐aligned, T1‐weighted images were segmented using recon‐all from FreeSurfer. Segmentations were improved using the enhanced white‐matter, pial‐surface contrast of the T2‐weighted sequence. Cortical thickness was calculated in native space (i.e., subject‐specific) at a spatial density of 0.9 mm intervertex distance. Such values are downsampled and reported at a spatial resolution that has an intervertex spacing of 2 mm. This space is termed “the standard grayordinate space” and has 32,492 vertices per hemisphere (Glasser et al., 2013).

##### Regions of interest and cortical thickness

Given the 64,984 vertices (32,492 per hemisphere), we calculated the average cortical thickness in 333 regions of interest (ROIs) based on a functional atlas (Gordon et al., 2016). Each ROI has a different number of vertices. The reported value for each ROI corresponds to the average of the cortical thickness values for the vertices defining each ROI. In addition, this functional atlas groups ROIs in 13 functional networks: Auditory (*n* = 24), Cingulo opercular (*n* = 40), Cingulo‐parietal (*n* = 5), Default (*n* = 41), Dorsal attention (*n* = 32), Fronto‐parietal (*n* = 24), Retrosplenial temporal (*n* = 8), Somatosensory‐lateral (*n* = 38), Somatosensory‐medial (*n* = 8), Salience (*n* = 4), Ventral attention (*n* = 23), Visual (n = 39). There are also 47 ROIs that were not assigned to any functional system. We used as predictors of improvement in dual‐task cost with exercise, the cortical thickness values from each functional network.

### 2.5 Statistical analyses

To investigate whether the improvement in dual‐task cost on gait speed in response to the ABC‐C exercise program differed between freezers (*n* = 23 for exercise‐first and *n* = 16 for exercise‐second) and nonfreezers (*n* = 21 for exercise‐first and *n* = 22 for exercise‐second), we used an independent *t*‐test. We also ran a repeated measures ANOVA test to compare differences in gait speed (single and dual‐task conditions) and dual‐task cost on gait speed having group (freezers and nonfreezers) and time points (baseline, midpoint, and final) as factors. Whenever a significant *F*‐value was obtained, post hoc comparisons (Tukey test adjustments) were used. Finally, we also controlled (ANCOVA test) for baseline differences between freezers and nonfreezers in dual‐task cost on gait speed, MDS‐UPDRS‐III score, disease duration, PIGD score, and gender (Table 1). The significance level for all analyses was set at *p <* .05.

**TABLE 1:** Characteristics of the 82 people with Parkinson's disease who we compared effects of the ABC‐C program on improvement in dual‐task cost on gait speed (mean ± SD)

Characteristics	Freezers (*n* = 39)	Nonfreezers (*n* = 43)	*p* value
Men/women (number)	30/9	24/19	n/a
Age (years)	68.1 (7.0)	69.2 (8.0)	.484
Height (cm)	175.3 (9.6)	172.4 (9.7)	.189
Body mass (kg)	80.5 (14.1)	77.9 (16.4)	.438
MoCA (score)	25.6 (3.8)	25.8 (3.2)	.821
Disease duration (years)	8.6 (5.5)	4.4 (3.7)	**<.001**
Hoehn and Yahr (a.u.)	2.3 (0.6)	2.1 (0.5)	.185
MDS‐UPDRS motor (score)	46.0 (12.7)	38.3 (10.2)	**<.001**
LEDD (mg/day)	820.3 (984.2)	535.3 (292.0)	.092
PIGD (score)	6.2 (2.9)	4.4 (2.3)	**.002**
PIGD subtype (%)	82.1	46.5	n/a
NFoGQ (score)	13.8 (5.5)	n/a	n/a
Dual‐task cost on gait speed (%)	−18.0 (10.1)	−13.9 (9.8)	**.030**

*Note:* Values in bold signifies that p‐value is less than .05. Abbreviations: a.u., arbitrary unit; LEDD, levodopa equivalent daily dose; MDS‐UPDRS‐III, Movement Disorders Society Unified Parkinson's Disease Rating Scale motor subscale; MoCA, Montreal Cognitive Assessment; n/a, not applicable; NFoGQ, new freezing of gait questionnaire; PIGD, postural instability and gait disorder.

To characterize associations between cortical thickness per functional network and responses to exercise using dual‐task cost on gait speed improvement, we used data from people with PD with high quality MRI data at baseline (*n* = 51), calculated the cortical thickness at 333 cortical areas, grouped those areas *per* functional system (Gordon et al., 2016), and assessed associations between responses to exercise and specific functional systems by calculating models per functional system to predict dual‐task cost of gait speed improvement as a function of cortical thickness independently for freezers, nonfreezers and combining freezers and nonfreezers. Improvement in dual‐task cost values were normalized using a logarithmic boxcox transformation (Adams, 2005) optimized by gradient descent. Models were calculated independently for each functional network using partial least squares regression (PLSR) (Rosipal, Kr, & Krämer, 2005) using MATLAB r2016b (The Mathworks Inc., Natick, MA). In PLSR, the redundancy of predictor variables is minimized by calculating a new set of predictor variables that maximizes outcome prediction. The new set of variables is calculated as a linear combination of the original predictor variables (Rosipal et al., 2005) and the modeler specify the number of components (or dimensions) used to project the data on. Increasing the number of components tends to increase within‐sample accuracy but results might not generalize. Here we decided to use four, a relatively low number of components in all the models, to decrease the likelihood of overfitting.

Additionally, to have a better estimation of the performance of the models on external datasets, models' goodness of the fit was determined using hold‐out cross validation, as we have done on similar studies (Miranda‐Dominguez et al., 2020; Rudolph et al., 2018). To do this, we reserved data from three participants to test performance of the models and used the remaining data to fit PLSR models. This approach was repeated 10,000 times when data from freezers and nonfreezers were combined (*n* = 51 unmatched; *n* = 42, matched for motor severity). When models were trained using data from freezers and nonfreezers independently, the number of repetitions was reduced to the maximum number of combinations that can be made given the corresponding sample size and reserving three participants out for cross validation. Concretely, this approach led to 1,330 distinct models for freezers, matched and unmatched since the two cases correspond to the same sample size (*n* = 21). For nonfreezers, we trained 1,330 models for unmatched data (*n* = 30) and 4,060 models for matched data (*n* = 21). Significance was calculated after comparing out‐of‐sample performance of the models trained with real data versus models trained with null‐hypothesis data. Null‐hypothesis data were created by permuting randomly values of dual‐task cost on gait speed improvement and cortical thickness within the people with PD used for modeling. For null‐hypothesis data, we trained 10,000 models using permuted data and tested the performance on unpermuted out‐of‐sample data (three participants out). Performance of the models was determined by the mean square errors of the predicted versus observed improvement in dual‐task cost on gait speed. In this study, we only report systems that predicted cortical thickness better than chance with an medium effect size (>0.50) calculated using Cohen's *d* (Cohen, 1988). Analyses were repeated after matching the original sample (*n* = 51) for motor severity (MDS‐UPDRS‐III), as higher motor severity is associated with higher disease severity (Tanji et al., 2008). We did not use the MDS‐UPDRS‐III motor scores at baseline as a covariate in the PSLR model due to lack of linearity, batches differences, and low correlation between MDS‐UPDRS‐III motor scores and the other variables (i.e., dual‐task cost on gait speed and cortical thickness). Thus, we decided to conduct our analysis using a high‐quality sample matched for this variable but also repeated the analyses using unmatched data.

To rule‐out the possibility that group differences in baseline cortical thickness could be driving our results, we ran a repeated measures ANOVA test to characterize differences in cortical thickness at baseline for the effects of networks (default, dorsal attention, fronto‐parietal, visual, and sensorimotor‐lateral), group (freezers and nonfreezers) and their interaction. This analysis was performed for matched and unmatched data. The significance level for this analysis was set at *p <* .5.

## 3 RESULTS

### 3.1 Participants

**FIGURE 1:**
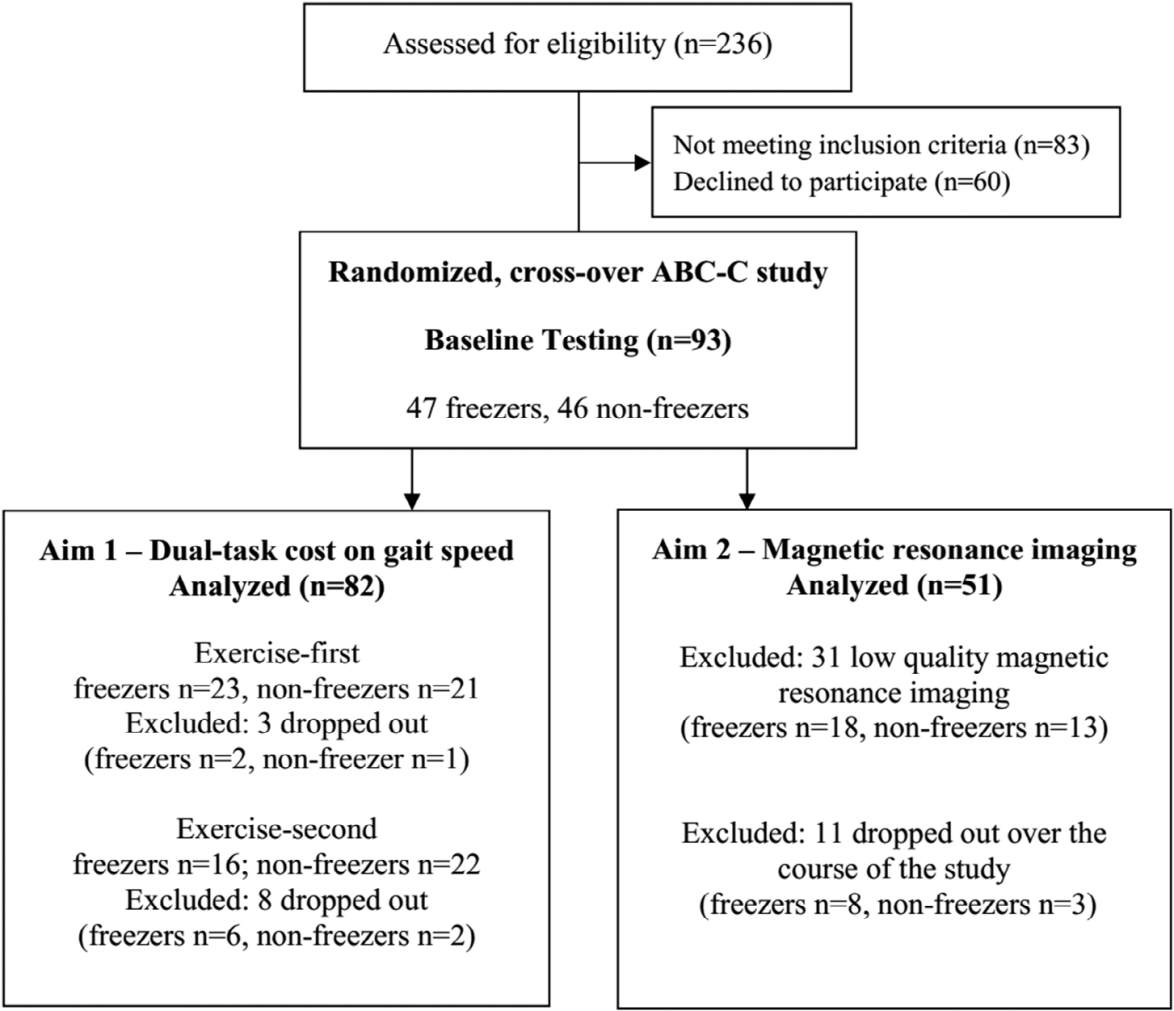
Consort diagram

The trial flow is presented in the consort diagram of Figure 1. There were 236 people with PD assessed for eligibility. Of these, 143 did not meet inclusion criteria and 60 declined to participate in the intervention, leaving 93 subjects consented and randomized into intervention groups (exercise and education programs). As the education program did not improve dual‐task cost on gait speed (Jung et al., in press) only people with PD who performed ABC‐C program were included in this analysis. A total of 82 people with PD who are freezers (*n* = 39) and nonfreezers (*n* = 43) were analyzed (Figure 1). Eleven people with PD (eight freezers and three nonfreezers) dropped out over the course of the study. Drop‐out reasons were: three sustained injuries outside of classes (heart attack, low‐back injury, sciatica), one had a medical procedure that prevented completing classes, one had a urinary tract infection with complications that led to falls, two did not want to commute to classes anymore, one changed medication, one subject declined intervention after baseline testing, and one was lost to follow up. In addition, one subject fell during exercise class resulting in a hip fracture (participant was spotted by the research assistant but fell while making a turn).

Characteristics of the freezer and nonfreezer groups are presented in Table 1. Freezers had greater disease severity (MDS‐UPDRS‐III score, PIGD, and duration of disease) and worse values of dual‐task cost on gait speed than nonfreezers (*p* < .05).

Of these 82 people with PD, we used a subset (21 freezers and 30 nonfreezers) who had high quality MRI at baseline to predict exercise response using dual‐task cost on gait speed improvement. Since freezers had greater motor severity than nonfreezers (*p* < .05), we also performed cortical thickness analysis as a factor to predict dual‐task cost on gait speed improvement after matching freezers (*n* = 21) and nonfreezers (*n* = 21) for motor severity in order to avoid our analysis to be biased by motor severity, as shown in Table 2.

**TABLE 2:** Characteristics of the people with Parkinson's disease who had high quality magnetic resonance imaging at baseline unmatched and matched for motor severity (mean ± SD)

	Unmatched			Matched for motor severity		
Characteristics	Freezers (*n* = 21)	Nonfreezers (*n* = 30)	*p* value	Freezers (*n* = 21)	Nonfreezers (*n* = 21)	*p* value
Men/women (number)	16/5	14/16	n/a	16/5	10/11	n/a
Age (years)	68.9 (7.5)	69.7 (9.2)	.735	68.9 (7.5)	69.8 (7.5)	.713
Height (cm)	173.6 (9.7)	169.7 (8.2)	.126	173.6 (9.7)	169.3 (8.3)	.126
Body mass (kg)	77.6 (11.5)	75.2 (16.1)	.570	77.6 (11.5)	74.3 (15.2)	.427
MoCA (score)	24.6 (4.5)	25.5 (3.6)	.439	24.6 (4.5)	25.1 (3.7)	.712
Disease duration (years)	9.1 (5.4)	5.0 (3.9)	**.002**	9.1 (5.4)	5.4 (4.1)	.014
Hoehn and Yahr (a.u.)	2.4 (0.7)	2.2 (0.6)	.424	2.4 (0.7)	2.4 (0.6)	1.000
MDS‐UPDRS motor (score)	45.2 (13.7)	38.2 (9.4)	**.034**	45.2 (13.7)	40.5 (9.1)	.191
LEDD (mg/day)	667.2 (319.6)	550.0 (286.6)	.195	667.2 (319.6)	547.6 (295.4)	.226
PIGD (score)	6.1 (3.2)	4.8 (2.4)	.117	6.1 (3.2)	5.3 (2.5)	.402
PIGD subtype (%)	85.7	56.7	n/a	85.7	61.9	n/a
NFoGQ (score)	13.5 (6.1)	n/a	n/a	13.5 (6.1)	n/a	n/a
Dual‐task cost on gait speed (%)	−18.1 (11.7)	−14.5 (11.1)	.301	−18.1 (11.7)	−15.6 (12.5)	.538

Abbreviations: a.u., arbitrary unit; LEDD, levodopa equivalent daily dose; MDS‐UPDRS‐III, Movement Disorders Society Unified Parkinson's Disease Rating Scale motor subscale; MoCA, Montreal Cognitive Assessment; n/a, not applicable; NFoGQ, new freezing of gait questionnaire; PIGD, postural instability and gait disorder.

### 3.2 Freezers showed a larger improvement in dual‐task cost on gait speed than nonfreezers

**FIGURE 2:**
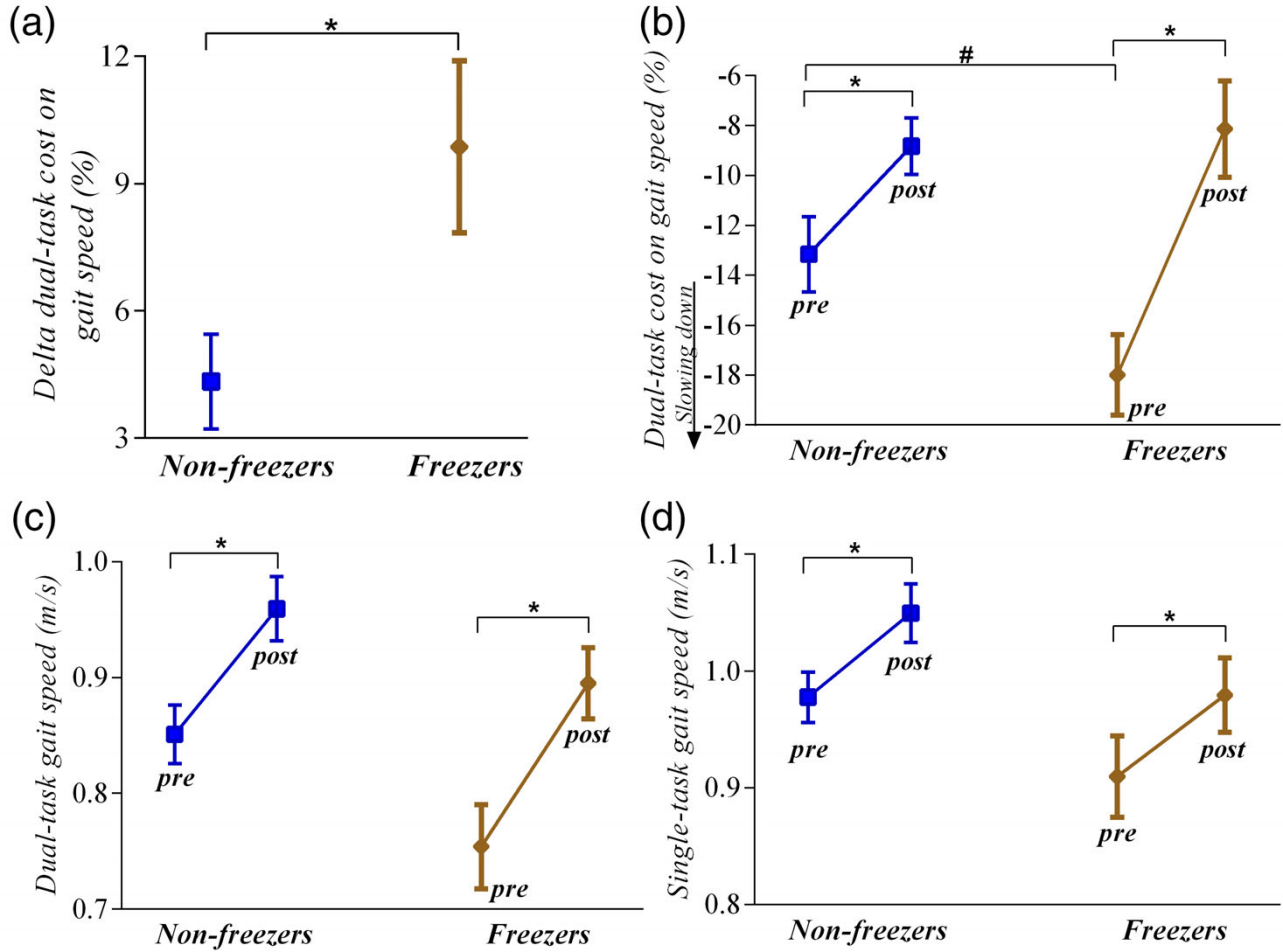
Mean ± *SE* for improvement (delta) in dual‐task cost on gait speed between nonfreezers (*n* = 43) and freezers (*n* = 39) after ABC‐C program (a). Freezers showed a larger improvement in dual‐task cost on gait speed than nonfreezers (*p* = .009). Mean ± SE for the dual‐task cost on gait speed (b), dual‐task gait speed (c), and single‐task gait speed (d) in the pre (baseline and midpoint values) and postexercise (midpoint and final values) for nonfreezers (*n* = 43) and freezers (*n* = 39). *Different from postexercise values (*p* < .05). ^#^Freezers showed worse dual‐task cost on gait speed than nonfreezers in the pre‐exercise (*p* = .030)

A larger improvement in dual‐task cost on gait speed were observed for freezers (9.8 ± 12.6%) compared to nonfreezers (4.3 ± 7.3%) following the ABC‐C program (mean difference = 6.3%, *t* = 2.6, *p* = .009) (Figure 2a).

### 3.3 Baseline differences did not influence on response to ABC‐C program in freezers

Freezers presented a larger improvement in dual‐task cost on gait speed relative to nonfreezers following ABC‐C program even after controlling for baseline differences in dual‐task cost on gait speed values (*F*_[1,78]_ = 0.18, *p* = .674), MDS‐UPDRS‐III scores (*F*_[1,78]_ = 2.23, *p* = .139), PIGD scores (*F*_[1,78]_ = 3.49, *p =* .065), disease duration (*F*_[1,78]_ = 0.13, *p =* .715), and gender (*F*_[1,78]_ = 1.71, *p =* .194).

### 3.4 Freezers and nonfreezers showed similar gait performance following ABC‐C program

Dual task‐cost on gait speed showed a significant group × time interaction (*F*_[1,80]_ = 5.99, *p =* .016). Post hoc comparisons showed that both freezers and nonfreezers improved dual‐task cost on gait speed at posttraining (*p* < .007), but freezers presented worse values of dual‐task cost on gait speed than nonfreezers at baseline (mean difference = 4.83%, *p* = .030; Figure 2b).

There was a significant time main effect for dual‐task gait speed (*F*_[1,80]_ = 28.94, *p* < .0001) and single‐task gait speed (*F*_[1,80]_ = 84.03, *p* < .0001), in which the posttest values were higher than the pretest values for both groups (Figure 2c,d).

### 3.5 Progression and attendance for the ABC‐C program

Class attendance in the ABC‐C program was similarly high in both groups, such that freezers and nonfreezers attended 92.6% and 94.2% of 18 classes, respectively. Freezers and nonfreezers showed similar progression of cognitive and physical difficulty across the six stations of ABC‐C program with the following percent of each group progressing to the most difficult levels: (1) Gait training (70.1 ± 13.5% and 71.0 ± 14.8%, respectively); (2) PWR!Moves® (54.5 ± 9.6% and 58.3 ± 8.9%, respectively); (3) Agility obstacle course (75.0 ± 11.3% and 77.5 ± 8.1%, respectively); (4) Lunges (70.2 ± 10.7% and 74.9 ± 7.4%, respectively); (5) Boxing (58.1 ± 10.0% and 60.3 ± 7.7%, respectively); and (6) Adaptive Tai Chi (48.0 ± 13.9% and 54.5 ± 12.3%, respectively).

### 3.6 Cortical thickness predicts dual‐task cost on gait speed improvement

Visual and fronto‐parietal cortical thicknesses were predictors of dual‐task cost on gait speed improvement only for freezers (Table 3; *n* = 21), whereas sensorimotor‐lateral cortical thickness was a predictor of dual‐task cost on gait speed improvement only for nonfreezers (Table 3; *n* = 30).

**TABLE 3:** Out‐of‐sample performance of partial least squares regression models predicting improvement in dual‐task cost on gait speed (dependent variable) as a function of cortical thicknesses (independent variable)

Groups	Network	ES	*R* ^2^	*p* value	*R* ^2^ dependent variable versus PLSR's first score
Unmatched					
All individuals (*n* = 51)	Sensorimotor‐lateral	0.54	0.35	<.001	0.50
	Fronto‐parietal	0.52	0.34	<.001	0.49
Freezers (*n* = 21)	Visual	0.77	0.49	<.001	0.67
	Fronto‐parietal	0.61	0.30	<.001	0.75
Nonfreezers (*n* = 30)	Sensorimotor‐lateral	0.55	0.25	<.001	0.76
Matched for motor severity					
All individuals (*n* = 42)	Visual	0.67	0.37	<.001	0.53
	Dorsal attention	0.55	0.30	<.001	0.53
	Fronto‐parietal	0.54	0.30	<.001	0.55
Freezers (*n* = 21)	Visual	0.77	0.49	<.001	0.67
	Fronto‐parietal	0.61	0.30	<.001	0.75
Nonfreezers (*n* = 21)	Visual	0.71	0.38	<.001	0.70
	Default	0.56	0.35	<.001	0.62

*Note:* Relationship of the dependent variable with the partial least squares regression (PSLR) first score (component).

Abbreviations: ES, effect size obtained after comparing out‐of‐sample prediction errors versus null data; *R* ^2^, correlation coefficient predicting out‐of‐sample improvement in dual‐task cost on gait.

We repeated this analysis after matching groups on MDS‐UPDRS‐III scores, which removed nonfreezer participants with the lowest MDS‐UPDRS‐III scores (*n* = 9). We observed that visual cortical thickness presented higher effect sizes than other areas and it was a common predictor of dual‐task cost on gait speed improvement for all subjects combined (freezers and nonfreezers) and for freezers and nonfreezers separately analyzed (Table 3; *n* = 21).

**FIGURE 3:**
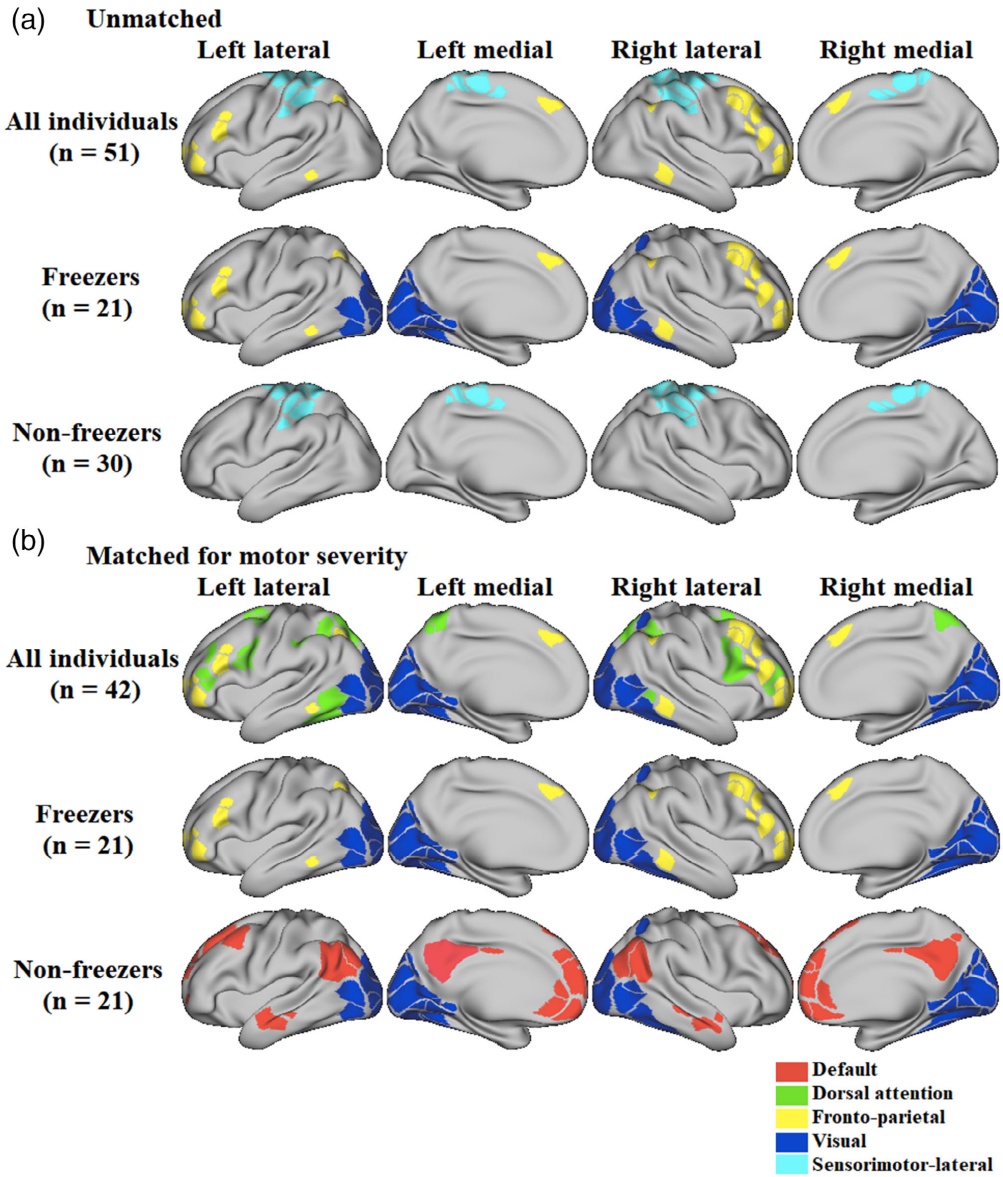
Cortical areas significantly associated with improvement in dual‐task cost on gait speed after 6 weeks of the Agility Boot Camp with Cognitive Challenge exercise program in all individuals, freezers and nonfreezers unmatched (a) and matched (b) for motor severity

Figure 3 illustrates the cortical areas that were predictors of dual‐task cost on gait speed improvement in all people with PD, freezers, and nonfreezers when unmatched (Figure 3a) and matched for motor severity (Figure 3b).

It is important to highlight that freezers (*n* = 21, 8.8 ± 10.5%) also showed a larger improvement in dual‐task cost on gait speed than nonfreezers (*n* = 30, 3.5 ± 5.1%) following the ABC‐C program in this subset of participants with imaging data (mean difference = 5.3%, *t* = 2.3, *p* = .0208). In addition, even when the freezer (*n* = 21) and nonfreezer (*n* = 21) groups were matched for motor severity, there was a strong trend (mean difference: 5.1%, *t* = 2.01, *p* = .051) toward a larger improvement in dual‐task cost on gait speed for freezers (8.8 ± 10.5%) compared to nonfreezers (3.6 ± 5.3%).

### 3.7 Cortical thickness at baseline is similar between freezers and nonfreezers

**FIGURE 4:**
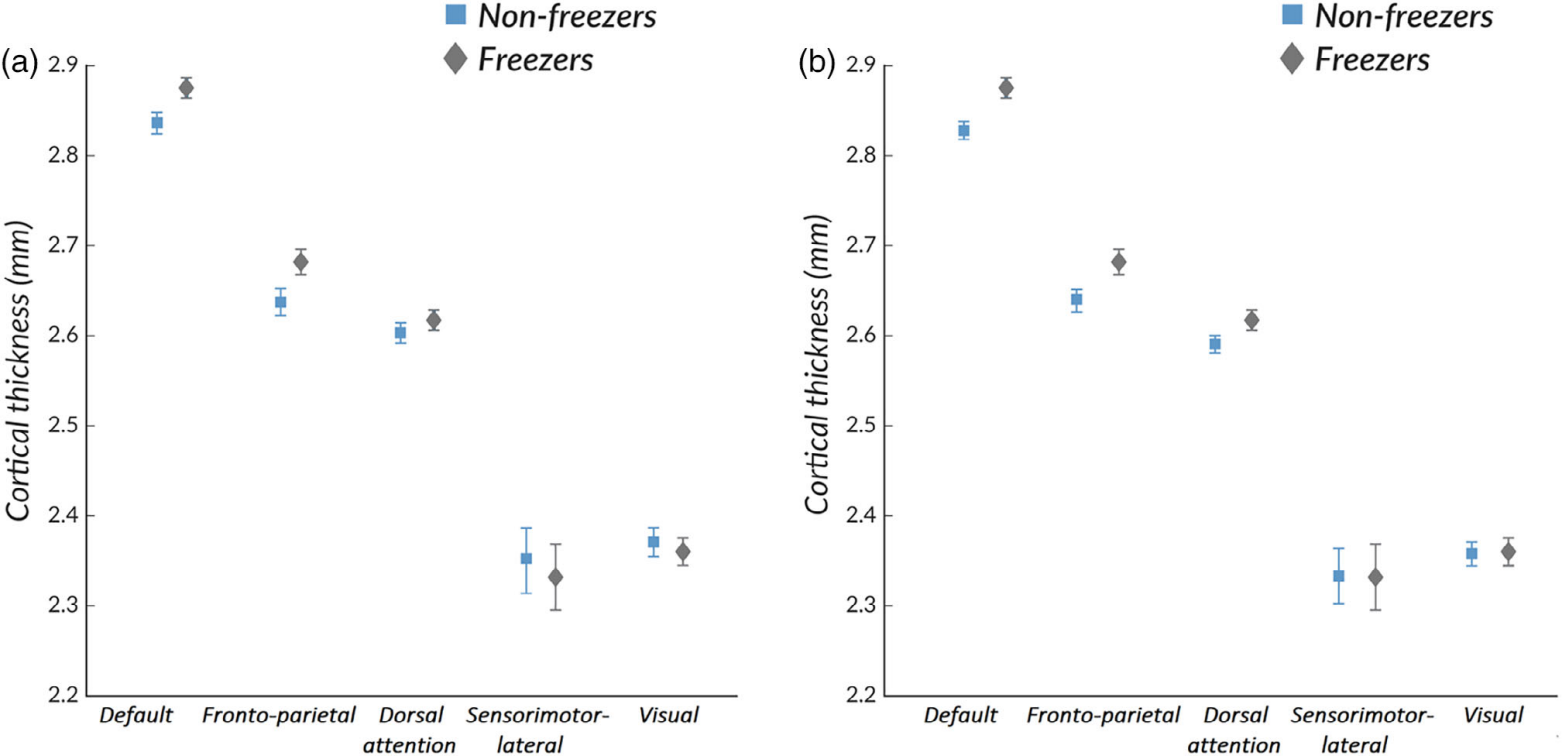
Mean ± *SE* for cortical thicknesses at baseline between nonfreezers and freezers unmatched (*n* = 30 and *n* = 21, respectively) (a) and matched (*n* = 21 and *n* = 21, respectively) (b) for motor severity

There was no significant group × networks effects on cortical thickness at baseline (*F*_[1,12]_ = 0.94, *p =* .51). As expected, we found significant differences in cortical thickness across networks using either unmatched (*F*_[1,12]_ = 11,765, *p* < .01) or matched (*F*_[1,12]_ = 9865.2, *p* < .01) data. In contrast, no differences in cortical thickness were found for group (*F*_[1,12]_ = 0.36, *p =* .55), as observed in Figure 4a,b, respectively. These results suggest our predictions of exercise responsiveness are unlikely to be driven by group differences in cortical thickness.

## 4 DISCUSSION

This is the first study to show that: (a) people with PD who are freezers show larger improvements in dual‐task cost on gait speed than nonfreezers from a cognitively‐challenging exercise program and (b) cortical thickness can predict the responsiveness to exercise on dual‐task cost on gait speed improvement in people with PD. Specifically, cortical thickness in areas related to FoG (visual cortex and fronto‐parietal cortex) can be used to predict responsiveness of freezers to cognitively‐challenging exercise. In addition, visual cortical thickness is a common predictor of responsiveness to cognitively challenging exercise in people with PD.

### 4.1 Dual‐task cost on gait speed improves more in freezers than nonfreezers

We were surprised to find that people with PD who are freezers showed larger improvements in dual‐task cost on gait speed with the ABC‐C exercise program than nonfreezers (Figure 2a). Previous studies have shown that freezers have less effective motor‐learning capacities (Paul et al., 2018), an exaggerated loss of automaticity (de Souza Fortaleza et al., 2017), and worse attentional control to perform dual‐task walking than nonfreezers (Ginis, Nackaerts, Nieuwboer, & Heremans, 2018). We also found that freezers had worse dual‐task cost than nonfreezers but they reduced their dual‐task cost even more than nonfreezers. This finding suggests that freezers can show even larger improvements of automaticity and/or reduced attentional control of gait compared to nonfreezers after cognitively‐challenging exercise.

The ABC‐C program incorporates a combination of motor and cognitive exercises performed simultaneously and repetitively, that are important characteristics to improve both the automaticity and attentional control in freezers (Peterson, King, Cohen, & Horak, 2016). In fact, a recent study also demonstrated that consecutive and integrated dual‐task training for 6 weeks lead to improvements in dual‐task gait speed in freezers (Strouwen et al., 2017). Since freezers benefited most from the ABC‐C program for dual‐task cost on gait speed, we conclude that cognitively‐challenging interventions, including dual‐task exercises can be effectively delivered to freezers to improve gait automaticity. Although baseline values of dual‐task cost and disease severity (MDS‐UPDRS‐III scores, PIGD scores, and disease duration) were worse in freezers, these baseline values did not influence the improvement in dual‐task cost on gait speed in response to exercise. Thus, further studies should consider specific exercise training (dual‐task, cognitively challenging exercises) to restore gait automaticity in freezers.

### 4.2 Cortical thickness predicts responsiveness to cognitively‐challenging exercise

No previous study has explored cortical factors associated with responsiveness to exercise in people with PD. Using cortical thickness at baseline as a predictor of response to exercise is important because a recent study demonstrated that baseline and 1‐year cortical thinning predicts the long‐term progression in motor and nonmotor symptoms of people with PD (Filippi et al., 2020). Thus, cortical thickness may be used not only for predicting disease progression but also to predict responsiveness to exercise. Only one study explored factors related to demographics, PD severity, physical and cognitive abilities, and perceived health as predictors of responsiveness of balance and gait speed among people with PD following a gait and balance training intervention; but the dependent variable was single‐task gait speed (Lofgren, Conradsson, Joseph, et al., 2019). As both the motor and cognitive tasks compete for cortical resources in dual‐task conditions (Wu & Hallett, 2005, 2008) and cortical atrophy is well documented in people with PD (Jha et al., 2015; Kostic et al., 2012), our novel findings demonstrate the importance of integrity of particular cortical areas for dual‐task cost on gait speed responsiveness to exercise in people with PD. However, we did not find differences in cortical thicknesses at baseline between our cohort of freezers and nonfreezers, although other studies have reported that freezers have more cortical atrophy than nonfreezers (Jha et al., 2015; Kostic et al., 2012).

A possible explanation for similar cortical thickness in freezers and nonfreezers in our study, but not some previous studies, is that cortical atrophy has been associated with cognitive dysfunction in freezers (Jha et al., 2015). However, freezers and nonfreezers in our study showed similar cognitive profile according to MoCA scores (Table 1) as well as similar cortical thickness (Figure 4), whereas in previous studies freezers showed worse cognitive performance and more cortical atrophy than nonfreezers (Jha et al., 2015; Kostic et al., 2012). Despite similar values of cortical thickness at baseline between freezers and nonfreezers, our study is the first to show that specific cortical areas at baseline were associated with larger improvements in dual‐task cost on gait speed following ABC‐C exercise program depending on freezing status and motor severity (i.e., when we matched for motor severity by excluding mild nonfreezers). However, future studies should consider more complex prediction models including cognitive function, motor, and nonmotor symptoms and MRI metrics, such as cortical thinning (Filippi et al., 2020; Sarasso, Agosta, Piramide, & Filippi, 2020), that might further improve the prediction of responsiveness to exercise in people with PD.

### 4.3 Visual and fronto‐parietal cortical thicknesses predicts dual‐task cost on gait speed improvement in freezers

We found that visual and fronto‐parietal cortical thicknesses at baseline predicted dual‐task cost on gait speed improvement following the ABC‐C program in freezers. Impairments in visual and fronto‐parietal cortical areas have been implicated in FoG. Studies have shown an association between reduced functional connectivity of the fronto‐parietal executive attention and visual cortical networks and the severity of FoG (Canu et al., 2015; Tessitore et al., 2012). Visual‐motor abnormalities are more common in freezers (Davidsdottir, Cronin‐Golomb, & Lee, 2005; Weil et al., 2016) and correlated with the severity of gait impairment (Uc et al., 2005). In addition, gray matter atrophy in the inferior parietal lobe in freezers is associated with deficits in dual‐task gait speed and FoG severity (Herman et al., 2014). The current study demonstrates that freezers who had larger visual and fronto‐parietal cortical thicknesses at baseline relative to other freezers were more likely to respond to cognitively‐challenging interventions. Thus, the visual and fronto‐parietal cortical thicknesses at baseline could determine if freezers will be responders or nonresponders to exercise interventions. Our results are important because freezers depend on visual‐attentional cues to perform dual‐task walking (Ginis et al., 2018), but they present dysfunction in cortical areas related to visual‐attentional process (Canu et al., 2015; Tessitore et al., 2012). Future studies should determine whether cognitively‐challenging interventions can enhance visual‐attentional networks to improve FoG and gait automaticity. Additionally, an interesting avenue of research would be to see if cortical thickness can be used as an outcome and if it is an outcome that is amenable to change following cognitively‐challenging interventions, such as ABC‐C program.

Only fronto‐parietal cortical thickness was a predictor of dual‐task cost on gait speed improvement in freezers, when groups were matched (or unmatched) for motor severity, but not in nonfreezers (Table 3). Our results reinforce the findings in the literature about fronto‐parietal areas having a significant role in FoG, given that abnormal gait‐posture coupling (Nutt et al., 2011), increased sensitivity to dual‐task effects during walking (de Souza Fortaleza et al., 2017), and alterations in attentional set‐shifting (Peterson et al., 2016) are factors associated with FoG and modulated by fronto‐parietal areas. Abnormal activity (Herman et al., 2014; Shine et al., 2011) and gray matter atrophy (Kostic et al., 2012) in fronto‐parietal regions are associated with FoG severity, suggesting a possible compensatory role of these areas to overcome freezing episodes during walking (Piramide et al., 2020; Shine et al., 2013). Therefore, we hypothesized that fronto‐parietal cortical thickness may play an important role in distinguishing freezers from nonfreezers and it might be the reason why freezers respond better to cognitively challenging exercise than nonfreezers in terms of gait automaticity (i.e., improved dual‐task cost on gait speed).

### 4.4 Sensorimotor‐lateral cortical thickness is a predictor of dual‐task cost on gait speed improvement in nonfreezers

Larger sensorimotor‐lateral cortical thickness at baseline was positively associated with improvement in dual‐task cost on gait speed in nonfreezers. People with PD have deficits in central sensorimotor integration (Lewis & Byblow, 2002) that affect walking performance. In fact, deficits in lower limb proprioception are significantly associated with fall incidence in people with PD (Paul et al., 2014). In addition, complex gait tasks, such as presented in the ABC‐C exercise program, result in increased activation within somatosensory cortical regions (Bradford, Lukos, & Ferris, 2016), consistent with a greater role for somatosensory information during dual‐task walking. The current study shows that nonfreezers who had larger sensorimotor‐lateral cortical thickness at baseline responded better to exercise.

When nonfreezers were matched for motor severity with the freezers, the default network cortical thickness (prefrontal and parietal areas) was a predictor of dual‐task cost on gait speed improvement in individuals with severe PD motor symptoms following the ABC‐C program. Previous studies have demonstrated increased prefrontal cortex activity during turning and walking in people with PD, consistent with a loss of automaticity (Belluscio, Stuart, Bergamini, Vannozzi, & Mancini, 2019). In addition, facilitation of dorsolateral prefrontal cortex with bilateral transcranial direct current stimulation reduces dual‐task cost on gait speed in people with PD (Swank, Mehta, & Criminger, 2016). Thus, future studies should determine whether longer‐lasting, cognitively‐challenging interventions can improve default cortical thickness, which is associated with improved gait automaticity (dual‐task cost on gait speed).

### 4.5 Visual cortical thickness is a common predictor of dual‐task cost on gait speed improvement

When freezers and nonfreezers were matched for motor severity by eliminating mild nonfreezers (*n* = 9), visual cortical thickness was a common predictor of dual‐task cost on gait speed improvement in all participants, with and without FoG. That is, the visual cortex appears to be particularly important in those with more severe PD and/or FoG. Although the dorsal attention and the fronto‐parietal cortical thicknesses also predicted dual‐task cost on gait speed improvement across all severity‐matched people with PD, visual cortical thickness presented the largest effect size for all subjects (0.67), freezers (0.77), and nonfreezers (0.71) when compared to other areas (Table 3).

The visual cortex may be especially important to compensate for impaired use of proprioception in people with severe PD (Lira et al., 2020; Weil et al., 2016). Although vibrotactile cues can reduce dual‐task cost on gait speed in individuals with PD (Stuart & Mancini, 2019), they showed a higher dependence on visual information for motor and postural control than normal and this dependence increases with severity of disease (Weil et al., 2016). Indeed, visual cueing improves walking and FoG in severe PD (Ginis et al., 2018). In addition, when people with PD reach or step without view of their limbs, they undershoot targets, although they are accurate when they can view both the target and their limbs (Jacobs & Horak, 2006). Visual abnormalities in people with PD are correlated with impairments in postural control, gait, and severity of the PD (Antal, Bandini, Keri, & Bodis‐Wollner, 1998). People with more severe PD likely depend upon visual information to perform dual‐task walking. Thus, our results demonstrated that people with severe PD who have larger visual cortical thickness at baseline relative to other people with severe PD are more likely to respond to cognitively‐challenging interventions in terms of dual‐task cost on gait speed improvement. In fact, we have previously demonstrated that 12 weeks of challenging exercises increased activation in visual temporal areas in freezers (Silva‐Batista et al., 2020). Thus, future studies should determine whether cognitively‐challenging interventions can enhance visual‐sensory networks to improve gait automaticity in freezers.

### 4.6 Limitations

This study has limitations. First, we had to exclude imaging data from 31 participants after a detailed inspection of neuroimaging, ending up with a reduced sample size of 51 brains. While this reduction decreased the power of the estimations, our curated sample was not contaminated by unrealistic estimates of cortical thickness, a known problem that has led to conflicting results in neuroimaging studies (Ducharme et al., 2016). In this modest number of participants, we estimated cortical thickness in 333 brain areas that were grouped into 13 functional systems (Gordon et al., 2016). To protect against false positives and over fitting, associations between cortical thickness and improvement in dual‐task cost on gait speed were estimated by predictive models, as opposed to correlations. Goodness of the models' fit were estimated using hold‐out cross validation and significance of the findings are reported using null‐hypothesis data generated by permutations. This robust approach suggests our results can be generalizable. Second, Gordon et al's classification (Gordon et al., 2016) was entirely based on young healthy controls with a mean age of ~25 years, as there is no such atlas for people with PD. Further studies are needed to characterize the reshaping of ROIs and networks in PD. However, we believe that this lack of accuracy might be damped by the well documented adaptive and compensatory cortical and subcortical changes in people with PD (Canu et al., 2015; Piramide et al., 2020; Shine et al., 2013; Tessitore et al., 2012). Third, imaging data were acquired using two different scanners, because the old scanner (Siemens Trio 3T) was replaced by the Siemens Prisma 3T scanner during the study. To control for this change, we calibrated the settings on the scanners with the same control subjects. In addition, there was no significant diagnosis × scanner on cortical thickness at baseline (data not shown) in matched or unmatched groups for motor severity. For this reason, no corrective measure was needed. This is not surprising given the fact that the measurement of anatomical features (i.e., cortical thickness) from T1 images is robust enough across scanners and manufacturers. Fourth, there was no washout period between the first and second intervention in this cross‐over design, so we could not rule out a possible carry‐over effects from the first interventions. However, carry‐over effects are unlikely because sequence and period effects were nonsignificant. Fifth, participants were assessed in their practical OFF‐state, only. Future studies should consider testing subjects both in the OFF and ON state, as dual‐task cost on gait may be affected by levodopa state. Sixth, our intervention was complex and highly challenging, thus, the results can be generalized only to exercise modalities that include both physical and attentional demands in subjects able to perform complex mobility tasks in a small group, without assistance.

## 5 CONCLUSIONS

People with PD who experience FoG benefited more from this cognitively challenging ABC‐C exercise program in terms of dual‐task cost on gait speed improvement. Visual and fronto‐parietal cortical thickness at baseline predicted which freezers responded to the ABC‐C program and may explain why freezers responded better than nonfreezers since these areas are more impaired in freezers. Finally, visual cortical thickness was a common predictor of responsiveness to this cognitively challenging exercise program consistent with the importance of visual compensation for impaired proprioception in people with motor severity in PD.

## CONFLICT OF INTEREST

The author(s) declared the following potential conflicts of interest with respect to the research, authorship, and/or publication of this article: Dr Horak has an equity interest in ERT‐APDM, a company that may have a commercial interest in the results of this research and technology. This potential conflict of interest has been reviewed and managed by the Research and Development Committee at the VA Portland Health Care System and the Institutional Review Board at Oregon Health & Science University. They have put in place a plan to help ensure that this research study is not affected by the financial interest.

## Supporting information


**Figure S1** Improvement in dual‐task cost on gait speed as a function of cortical thickness for each ROI for freezers (Fr) and nonfreezers (NF). Def = default; DoA = dorsal attention; FrP = fronto‐parietal; Vis = visual; SMI = sensorimotor‐lateral.
**Figure S2**. Improvement in dual‐task cost on gait speed as a function of mean cortical thickness for each network for freezers (Fr) and nonfreezers (NF). Def = default; DoA = dorsal attention; FrP = fronto‐parietal; Vis = visual; SMI = sensorimotor‐lateral.
**Figure S3**. Betaweights for each network, in all individuals, freezers and nonfreezers unmatched and matched for motor severity. Def = default; DoA = dorsal attention; FrP = fronto‐parietal; Vis = visual; SMh = sensorimotor‐lateral.
**Figure S4**. Cortical thicknesses at baseline between freezers (Fr—red) and nonfreezers (NF—blue) unmatched (A) and matched for motor severity (B). Def = default; DoA = dorsal attention; FrP = fronto‐parietal; Vis = visual; SMI = sensorimotor‐lateral.
**Table S1**. Relationship of the dependent variable (dual‐task cost on gait speed improvement) with the predictors (cortical thickness) and partial least squares regression (PSLR) first score (component).

## Data Availability

Data sharing is not applicable to this article as no new data were created or analyzed in this study.
