# Supplementary material for: Cortical thickness as predictor of response to exercise in people with Parkinson's disease
Source: Hum Brain Mapp. 2020 Oct 9;42(1):139–53. doi: 10.1002/hbm.25211 (PMC7721225; doi:10.1002/hbm.25211)
Supplement: Supplementary file 1 — Figure S1 Improvement in dual‐task cost on gait speed as a function of cortical thickness for each ROI for freezers (Fr) and nonfreezers (NF). Def = default; DoA = dorsal attention; FrP = fronto‐parietal; Vis = visual; SMI = sensorimotor‐lateral. Figure S2. Improvement in dual‐task cost on gait speed as a function of mean cortical thickness for each network for freezers (Fr) and nonfreezers (NF). Def = default; DoA = dorsal attention; FrP = fronto‐parietal; Vis = visual; SMI = sensorimotor‐lateral. Figure S3. Betaweights for each network, in all individuals, freezers and nonfreezers unmatched and matched for motor severity. Def = default; DoA = dorsal attention; FrP = fronto‐parietal; Vis = visual; SMh = sensorimotor‐lateral. Figure S4. Cortical thicknesses at baseline between freezers (Fr—red) and nonfreezers (NF—blue) unmatched (A) and matched for motor severity (B). Def = default; DoA = dorsal attention; FrP = fronto‐parietal; Vis = visual; SMI = sensorimotor‐lateral. Table S1. Relationship of the dependent variable (dual‐task cost on gait speed improvement) with the predictors (cortical thickness) and partial least squares regression (PSLR) first score (component). [file HBM-42-139-s001.docx]

**Supplementary File – Figures and Tables**


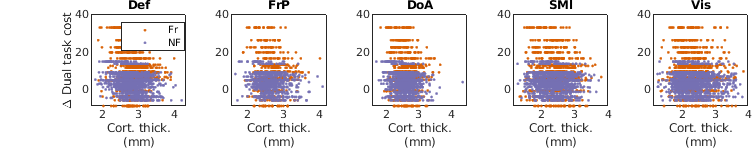


**Figure 1.** Improvement in dual-task cost on gait speed as a function of cortical thickness for each ROI for freezers (Fr) and non-freezers (NF). Def = Default; DoA = Dorsal attention; FrP = Fronto-parietal; Vis = Visual; SMI = Sensorimotor-lateral.


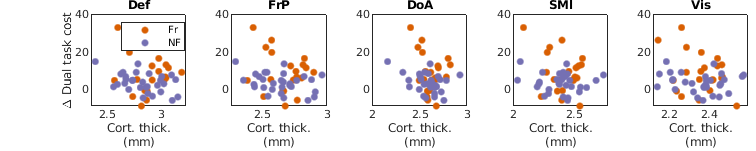


**Figure 2.** Improvement in dual-task cost on gait speed as a function of mean cortical thickness for each network for freezers (Fr) and non-freezers (NF). Def = Default; DoA = Dorsal attention; FrP = Fronto-parietal; Vis = Visual; SMI = Sensorimotor-lateral.


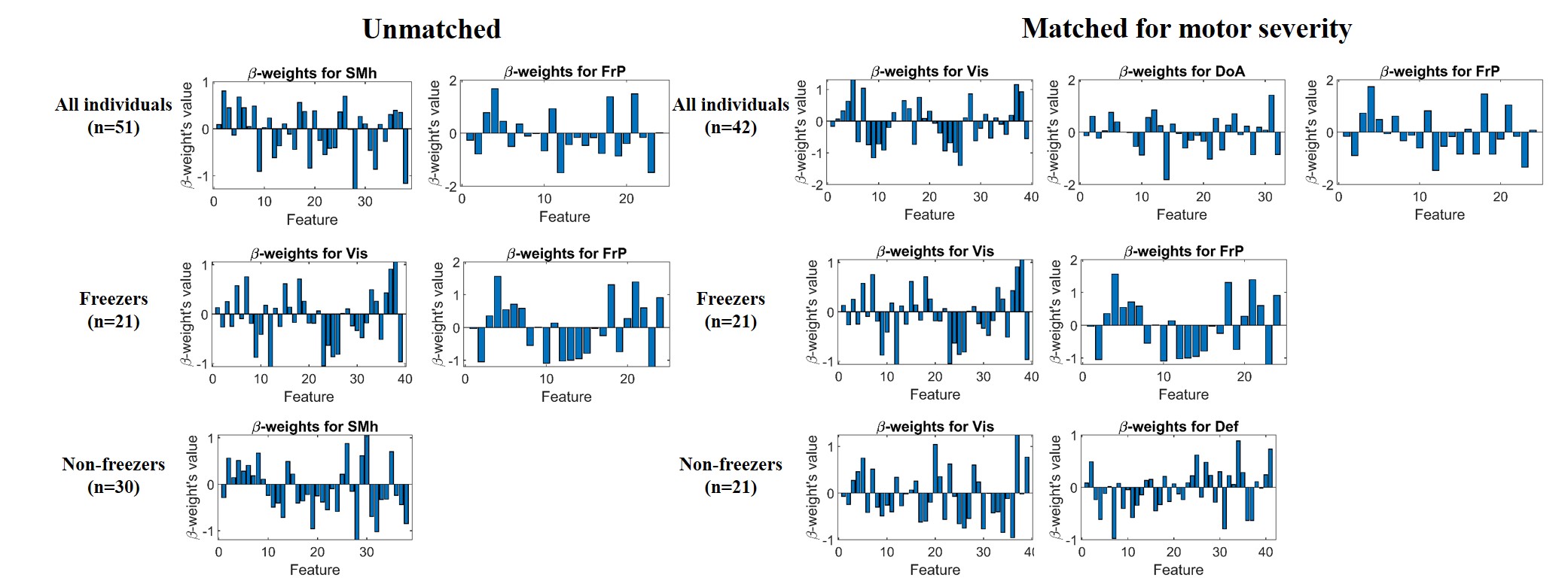


**Figure 3**. Betaweights for each network, in all individuals, freezers and non-freezers unmatched and matched for motor severity. Def = Default; DoA = Dorsal attention; FrP = Fronto-parietal; Vis = Visual; SMh = Sensorimotor-lateral.

**Figure 4.** Cortical thicknesses at baseline between freezers (Fr - red) and non-freezers (NF -blue) unmatched (A) and matched for motor severity (B). Def = Default; DoA = Dorsal attention; FrP = Fronto-parietal; Vis = Visual; SMI = Sensorimotor-lateral.

Thick lines and circles indicate interquartile range and median, respectively.

**Table 1.** Relationship of the dependent variable (dual-task cost on gait speed improvement) with the predictors (cortical thickness) and partial least squares regression (PSLR) first score (component).

| **Groups** | **Network** | **R^2^ dependent variable versus cortical thickness** | **R^2^ dependent variable versus PLSR’s 1^st^ score** |
| --- | --- | --- | --- |
| **Unmatched** |  |  |  |
| *All individuals (n=51)* | Sensorimotor-lateral | -0.17 | 0.50 |
|  | Fronto-parietal | -0.18 | 0.49 |
|  |  |  |  |
| *Freezers (n=21)* | Visual | -0.36 | 0.67 |
|  | Fronto-parietal | -0.19 | 0.75 |
|  |  |  |  |
| *Non-freezers (n=30)* | Sensorimotor-lateral | -0.12 | 0.76 |
|  |  |  |  |
| **Matched for motor severity** |  |  |  |
| *All individuals (n=42)* | Visual | -0.33 | 0.53 |
|  | Dorsal attention | -0.24 | 0.53 |
|  | Fronto-parietal | -0.18 | 0.55 |
|  |  |  |  |
| *Freezers (n=21)* | Visual | -0.36 | 0.67 |
|  | Fronto-parietal | -0.19 | 0.75 |
|  |  |  |  |
| *Non-freezers (n=21)* | Visual | -0.30 | 0.70 |
|  | Default | -0.51 | 0.62 |
